# COVID-19 und Weiterbildung – Überblick zu Forschungsbefunden und Desideraten

**DOI:** 10.1007/s40955-021-00190-7

**Published:** 2021-12-03

**Authors:** Anika Denninger, Bernd Käpplinger

**Affiliations:** Professur für Weiterbildung, Justus-Liebig-Universität Gießen, Karl-Glöckner-Str. 21B, 35394 Gießen, Deutschland

**Keywords:** COVID-19, Pandemie, Weiterbildung, Krise, Desiderate, Digitalisierung, E‑Learning, COVID-19-pandemic, Continuing education, Pandemic, Crisis, Desiderata, Digitalization, E‑learning

## Abstract

Der Beitrag gibt einen strukturierten Überblick zu verschiedenen Studien im Zeitraum von 2020 bis Mitte 2021 zur COVID-19-Pandemie und deren Auswirkungen auf die Erwachsenen- und Weiterbildung. Studientypen bzw. Publikationen werden unterschieden und wesentliche Befunde skizziert. Es werden Forschungsbedarfe und -lücken aufgezeigt sowie Impulse für noch differenziertere und vertiefende Analysen gegeben.

## Abstract (en)

The article provides a structured overview of various studies in the period from spring 2020 to mid-2021 that examined the impact of the COVID-19-pandemic on continuing education. Study types and publications are distinguished and key findings are outlined. Research needs and gaps are identified. Impulses for even more differentiated and in-depth-analyses are given.

## Einleitung

Krisenzeiten sind Lernzeiten. In der Geschichte hat sich oft gezeigt, dass gesellschaftliche, politische oder wirtschaftliche Umbrüche die Bedeutung der Erwachsenenbildung erhöhten. Erwachsenen- und Weiterbildung wird als Instrument zur Krisenbewältigung eingesetzt, was nicht unproblematisch ist. Nicht jedes gesellschaftliche Problem ist allein durch Bildung zu lösen. In kritischen Diskursen wird dies hinterfragt, was hier nicht vertieft wird. Trotzdem ist es naheliegend, da der Umgang mit Transitionen ein konstituierendes Merkmal der Erwachsenenbildung darstellt. So unterscheidet Schulenberg (1973) in kompensatorische, komplementäre und transitorische Erwachsenenbildung. Wie noch im Text gezeigt werden wird, werden die aktuellen Entwicklungen zum einen als Disruption begriffen und zum anderen als Beschleunigung von Transitionen, die sich schon vor Corona entwickelten. Beim letzten Punkt sei vor allem an die Digitalisierung gedacht. Mit der Corona-Pandemie hat sich seit 2020 insgesamt viel verändert, wobei wir noch nicht wissen, was davon dauerhaft („Neue Normalität“) oder was lediglich Momentaufnahme in einer Ausnahmesituation bleiben wird. Allerdings wiederholt sich die Geschichte nicht. In den Jahren 2020 und 2021 war die Erwachsenen- und Weiterbildung – anders als während der Flüchtlingskrise rund um das Jahr 2015 – nicht primär Nutznießer, sondern vor allem von der Krise betroffen (vgl. Tenorth 2020) und durch die Pandemie-Gegenmaßnahmen in ihrer Arbeit deutlich eingeschränkt.

Dieser Artikel soll einen Beitrag dazu leisten, die vorhandene und in der Entstehung befindliche Studienlage zu den Folgen der Pandemie auf die Weiterbildung besser zu überblicken und etwaige Forschungslücken zu identifizieren. Es wurden Recherchen zu Suchbegriffen wie „Corona“, „Covid“ und „Pandemie“ in einschlägigen Datenbanken wie FIS-Bildung vorgenommen, einschlägige Zeitschriften^1^ und periodische Erhebungen der Weiterbildung ausgewertet, Abfragen über wissenschaftliche Fachgesellschaften^2^ getätigt sowie Kolleginnen und Kollegen kontaktiert und befragt, um aktuelle Studien zu finden, die noch nicht in Datenbanken erfasst sind. Ferner wurde darauf geachtet, über eigene persönliche Netzwerke hinaus zu recherchieren, wenngleich die Erfassung natürlich eine Momentaufnahme darstellt und die Erfassung über Datenbanksysteme wie FIS-Bildung nicht komplett ersetzen kann. Spätere Forschungsprojekte können hieran andocken und etwaige, noch vorhandene Leerstellen füllen. Der Fokus der Recherche lag dabei auf Deutschland. Dies hat pragmatische Gründe, spiegelt aber gleichzeitig den bisherigen Verlauf des Umgangs mit der Pandemie wider, denn auch in vielen anderen Bereichen wurde verstärkt national statt international agiert und entschieden.

Die pandemiebegleitende Weiterbildungsforschung hat im Verlauf des ersten Jahres der Pandemie eine Reihe an Studien und Erhebungen hervorgebracht. Um einen Überblick darüber zu gewinnen, werden nachfolgend zunächst qualitative und quantitative Studien aus der Wissenschaft einerseits sowie Erhebungen von Verbänden andererseits vorgestellt und ein exemplarischer Blick auf die Ergebnisse geworfen. Zudem wird aufgezeigt, zu welchen laufenden wissenschaftlichen Studien in den nächsten Monaten weitere Ergebnisse zu erwarten sind. Zu betonen ist, dass es sich bei dem Forschungsüberblick um eine Momentaufnahme handelt und daher kein Anspruch auf Vollständigkeit gegeben ist. Darauf aufbauend werden zentrale Ergebnisse dieser Recherche synoptisch und analytisch zusammengefasst sowie etwaige Forschungslücken identifiziert. Schließlich werden die Effekte der Pandemie auf die Weiterbildung mit den drei Metaphern „Disruptor“, „Katalysator“ und „Brennglas“ beschrieben.

## Studien aus der Wissenschaft

Im Verlauf des ersten Jahres der COVID-19-Pandemie entstand national eine Reihe von wissenschaftlichen^3^ Studien, die sich mit den Auswirkungen der Pandemie auf den Weiterbildungssektor und der aktuellen Situation der Einrichtungen beschäftigen.^4^ Tab. 1 listet Studien auf und gibt einen Überblick über Erhebungszeiträume, Methodiken, Stichprobengrößen sowie Analysefokusse der Studien.

Der Unterscheidung nach quantitativer und qualitativer Forschung folgend werden im Weiteren wesentliche Ergebnisse der ausgewählten Studien überblicksartig vorgestellt.

**Table 1:** 

	Erhebungszeitraum	Studie	Methodik	Stichprobe	Analysefokus
Quantitativ	April 2020	Blitzbefragung des mmb Instituts (Schmid und Goertz 2020)	*Keine Details veröffentlicht*	64 Anbieter digitaler Lernservices und -tools	Effekte der Pandemie auf Anbieter der digitalen Bildungswirtschaft
	April & Mai 2020	IW-Covid-19 Panel (Flake et al. 2020)	Panel Fragebogen (monatlich)	555 (Welle 1) & 285 (Welle 2) Betriebe	Rolle der Weiterbildung in Betrieben während der Krisenzeit
	Mai 2020–Dez. 2021	IAB Sondererhebung Betriebe in der Covid-19-Krise (Bellmann et al. 2020)	Panel quant. Interviews (dreiwöchentlich)	1500–2000 Betriebe je Welle	Situation von Betrieben in der Corona-Krise
	Juni–Aug. 2020	wbmonitor Umfrage 2020 (Christ et al. 2021)	Panel Fragebogen (jährlich)	1925 WB-Anbieter	Auswirkungen der Pandemie auf die Weiterbildungsanbieter
Qualitativ	April–Mai 2020	Delphi-Studie zu Effekten der Pandemie auf die EB (Käpplinger und Lichte 2020)	Delphi	27 internationale Expert*innen für EB (Welle 1)	Unmittelbare und erwartete Effekte der Covid-19-Pandemie auf die EB
	Mai–Juni 2020	Befragung der Volkshochschulen in Rheinland-Pfalz (Rohs 2020)	Telefonisch leitfadengestützte Interviews	10 VHSen in Rheinland-Pfalz	Pandemiebedingte Einschränkungen in VHSen
	Okt.–Dez. 2020	Best Practices für neue Geschäftsmodelle und Programmanpassungen der Erwachsenenbildung (Grotlüschen und Weis 2021)	Telefon- & Video-Call-Interviews	13 Praktiker*innen der beruflichen Weiterbildung	Veränderungsprozesse in WB-Einrichtungen in der Pandemie
	Mai–Juni 2020	Befragung zu den Herausforderungen des pandemiebedingten Social Distancing im Bereich Alphabetisierung und Grundbildung (Koppel und Langer 2020)	Leitfadengestützte Interviews	4 Schnittstellenpersonen der Alphabetisierung und Grundbildung	Auswirkungen der Pandemie und des Social Distancing im Bereich Alphabetisierung und Grundbildung

### Quantitative Studien im Überblick

Drei der quantitativ angelegten Studien beschäftigen sich mit den Auswirkungen der COVID-19-Pandemie auf das Segment der betrieblichen Weiterbildung. Die im April 2020 durchgeführte Blitzbefragung des privaten mmb Instituts in Essen bei E‑Learning-Unternehmen geht der Frage nach, ob bzw. inwiefern die E‑Learning-Branche zu den „großen Profiteuren“ (Schmid und Goertz 2020) der COVID-19-Krise zählt. Die Ergebnisse der Umfrage zeigen ein differenziertes Bild. Während 48 % der 64 befragten E‑Learning-Unternehmen von einem gleichbleibenden Umsatz berichten, melden 25 % einen Umsatzrückgang und 27 % einen Umsatzzuwachs. Gleichzeitig rechnen 21 % der Unternehmen mit einer Aufstockung des Personals, während 29 % einen Personalabbau und 50 % keine Veränderungen planen. Den Ergebnissen nach spaltet die Krise „die digitale Bildungswirtschaft in nahezu gleich viele Gewinner und Verlierer – wobei für die meisten derzeit noch keine nennenswerte Veränderung wahrnehmbar ist“ (ebd.). Dennoch herrscht mit Blick auf die Zeit nach der Pandemie Optimismus unter den E‑Learning-Unternehmen, da die Krise aus Sicht der Mehrheit der befragten Unternehmen die Vorteile des E‑Learnings hervorhebe (98 %) und kein Rückfall in die bisherigen analogen Lerngewohnheiten (79 %) erwartet werde (vgl. ebd.).

Das IW-Covid-19-Panel des arbeitgebernahen Instituts der deutschen Wirtschaft (IW) widmet sich der Rolle der Weiterbildung in Unternehmen während der Krisenzeit. Die Ergebnisse der Unternehmensbefragung zeigen, dass die Krise verschiedene Auswirkungen auf die betriebliche Weiterbildung der 377 teilnehmenden Unternehmen hat. So hatten 44,5 % der befragten Unternehmen ihre Weiterbildungsaktivitäten zum Zeitpunkt der Befragung (Mai 2020) nicht verändert, während 25,9 % von einem Rückgang und 12 % von einem Anstieg berichten. Insgesamt verzeichnet die Befragung keinen generellen Einbruch der betrieblichen Weiterbildungsaktivitäten. Vielmehr wurde versucht, den pandemiebedingten Einbruch im Bereich der Präsenzveranstaltungen mittels digitaler Angebote abzufedern, was bei einem Drittel der laufenden und geplanten Weiterbildungsaktivitäten gelungen scheint. Es zeigte sich: Je kleiner das Unternehmen, desto seltener wurden alternative digitale Weiterbildungsangebote genutzt und desto größer ist der Informationsbedarf zu E‑Learning-Angeboten (vgl. Flake et al. 2020, S. 1–3).

Für die öffentliche Diskussion und den politischen Prozess zum Umgang mit den wirtschaftlichen Folgen der Corona-Krise mit wissenschaftlichen Daten hat das Institut für Arbeitsmarkt- und Berufsforschung (IAB) eine Sondererhebung gestartet. Die Studie „Betriebe in der Covid-19-Krise“ ist eine Erhebung im Drei-Wochen-Takt mit jeweils mehr als 1500 Betrieben zu ihrer Situation in der Corona-Krise und ihrem Umgang damit. Hier berichten zum Beispiel sechs von zehn weiterbildenden Unternehmen, dass sie im Jahr 2020 geplante oder begonnene Weiterbildungen abgesagt haben. Die Kontaktbeschränkungen sind der mit Abstand häufigste Grund dafür. 44 % der weiterbildenden Betriebe haben E‑Learning deutlich stärker genutzt als vor der Krise und ein gutes Drittel hat diese Lernform in der Krise neu eingeführt. Nur jeder zehnte Betrieb, der das Instrument der Kurzarbeit nutzt, verwendet ausgefallene Arbeitszeit, um seine Beschäftigten weiterzubilden. 81 % der befragten Unternehmen verzichten auf eine Weiterbildung während der Kurzarbeit, weil nicht absehbar sei, wann sie ihre Geschäftstätigkeit wieder in vollem Umfang aufnehmen könnten. Knapp 40 % erachten Weiterbildung als nachrangig in der Krise. Die Autorinnen und Autoren kommen zu dem Fazit: „Insgesamt ist festzuhalten, dass weniger Betriebe unter den gegenwärtigen Bedingungen in die Qualifizierung ihrer Beschäftigten investieren“ (Bellmann et al. 2020, S. 7).

Die größte jährlich wiederkehrende Umfrage bei Weiterbildungsanbietern in Deutschland ist der „wbmonitor“. Die Umfrage 2020 präsentiert Ergebnisse zu den Auswirkungen der Corona-Pandemie auf die Weiterbildungsanbieter von Frühjahr bis Sommer 2020. Die Ergebnisse zeigen, dass viele mit Beginn des Lockdowns im März 2020 ihre Präsenzkurse absagen mussten. Eine Umstellung auf virtuelle Formate brachte einen deutlichen Mehraufwand für die Einrichtungen mit sich und erfolgte bei rund vier von zehn laufenden Angeboten (vgl. Christ et al. 2021, S. 18). Daher wurden bei den befragten 1925 Anbietern insgesamt 77 % der geplanten Angebote verschoben oder gänzlich abgesagt (vgl. ebd., S. 7). Nach Beendigung des Lockdowns war die Realisierung von Präsenzveranstaltungen aufgrund behördlicher Auflagen von zahlreichen Veränderungen, wie z. B. geringere Teilnehmendenzahlen und strengen Hygienevorschriften, geprägt. Dies führte zu erheblichen finanziellen Verlusten bei den Anbietern (vgl. ebd., S. 22). Des Weiteren liefert die Umfrage Informationen zu den pandemiebedingten Auswirkungen auf die personelle und wirtschaftliche Situation der befragten Weiterbildungsanbieter. So mussten 40 % der Anbieter für ihre Beschäftigten Kurzarbeit anmelden. Betriebsbedingte Kündigungen wurden bei 5 % der Anbieter ausgesprochen. Zudem wird von 81 % der Anbieter angemerkt, dass die Situation ihre Mitarbeitenden, unabhängig der Anstellungsart, psychisch belastet. Eine wirtschaftliche Not ihrer Honorarkräfte identifizierten 70 % der Anbieter (vgl. ebd., S. 30–39). Um die wirtschaftlichen Auswirkungen der COVID-19-Krise zu verringern, bezogen die Anbieter staatliche Hilfen, wie z. B. Kurzarbeitergeld (34 %) und Corona-Soforthilfe (24 %) (vgl. ebd., S. 26). Die Einschätzung der wirtschaftlichen Stimmungslage der Weiterbildungsanbieter fiel verhalten aus und lieferte für das Jahr 2020 mit einem Wert von −13 erstmals seit Beginn der Messung im Jahr 2008 einen negativen Klimawert. Dieser liegt 57 Punkte unter dem Vorjahreswert. Mit Blick auf die Anbietersegmente, unterschieden nach Hauptfinanzierungsquellen, zeigen sich allerdings deutliche Differenzen. So verzeichnen die Anbieter des privat finanzierten Weiterbildungsbereichs mit einem Wert von −24 den schlechtesten Klimawert. Anbieter, die primär von den Arbeitsagenturen finanziert werden, verzeichneten dagegen einen positiven Klimawert von +6 (vgl. ebd., S. 11–15).

#### Quantitative Studien in der Entstehung

Neben den dargestellten Erhebungen sind für mehrere quantitative Studien noch Ergebnisse zu erwarten. So wurden bzw. werden gegenwärtig Daten für das Jahr 2020 seitens des „Adult Education Survey“ (AES) und des „Continuing Vocational Training Survey“ (CVTS) zur individuellen und betrieblichen Weiterbildungsbeteiligung erhoben und ausgewertet. Diese Erhebungen werden im Laufe der nächsten Monate bzw. Jahre Informationen liefern. Da es sich um regelmäßig durchgeführte Erhebungen handelt, werden sie interessante Zeitreihenvergleiche ermöglichen, wenngleich es keine Panelstudien sind, wie z. B. das „Nationale Bildungspanel“ (NEPS), das „Sozioökonomische Panel“ (SOEP) sowie das „IAB-Betriebspanel“, deren Ergebnisse mit Blick auf die Corona-bedingten Veränderungen in Zukunft zu verfolgen sind. Ebenfalls offen sind die Ergebnisse des „DIECovidSurvey“, der seit Oktober 2020 vom Deutschen Institut für Erwachsenenbildung (DIE) und dem Deutschen Volkshochschul-Verband (DVV) erarbeitet wird. Die Umfrage stellt eine Corona-Zusatzbefragung zur DIE-Anbieterstatistik dar und fokussiert die Auswirkungen der COVID-19-Pandemie auf die Volkshochschulen (vgl. DIE 2021).

### Qualitative Studien im Überblick

Neben den genannten quantitativen Erhebungen wurden und werden qualitative Studien zu den Auswirkungen der COVID-19-Pandemie im Bereich der Erwachsenenbildung durchgeführt. In der „Delphi-Studie“ von Käpplinger und Lichte (2020) von der Justus-Liebig-Universität Gießen wurden in der ersten Befragungsstufe im Frühjahr 2020 insgesamt 54 Wissenschaftlerinnen und Wissenschaftler der Erwachsenenbildung aus 19 Ländern bezüglich unmittelbarer und erwarteter Effekte der behördlichen Maßnahmen gegen COVID-19 auf die Erwachsenenbildung befragt (vgl. ebd., S. 780 f.). Für die meisten Befragten befindet sich die Erwachsenenbildung zum Zeitpunkt der Befragung aufgrund der pandemiebedingten Angebotseinbrüche weltweit in einer disruptiven Krise. Als besonders von der Disruption betroffen werden zum einen Benachteiligte betrachtet, die einen schlechten Zugang zu digitalen Lernumgebungen haben. Ferner werden Freiberuflerinnen und Freiberufler, selbstständige Erwachsenenbildnerinnen und Erwachsenenbildner sowie gemeinnützige Einrichtungen insbesondere hinsichtlich ihrer Finanzierung als besonders gefährdet eingestuft (vgl. ebd., S. 782–785). Für die Zukunft wird ein Anstieg des digitalen Lernens erwartet. Außerdem werden eine zunehmende Verschlechterung der Finanzierung der Erwachsenenbildung sowie mehr Ungleichheit bezüglich benachteiligter Gruppen prophezeit (vgl. ebd., S. 785–787). Die COVID-19-Pandemie wird sowohl als „Katalysator“ als auch „Brennglas“ beschrieben. Als Katalysator fungiert die Pandemie, indem sie bereits begonnene Entwicklungen im Bereich digitales Lernen massiv beschleunigt. Im Sinne eines Brennglases führt sie zu einer besseren Sichtbarkeit bestehender Probleme der Erwachsenenbildung, wie Teilsegmente prekärer Arbeit von manchen Beschäftigten in der Weiterbildung (vgl. ebd., S. 789 f.). Darüber hinaus wird aus einigen Ländern berichtet, dass autoritäre Regime die Pandemie für den Abbau von Freiheitsrechten und die Schwächung oppositioneller Kräfte zu nutzen versuchen.

Eine qualitative Studie zu den pandemiebedingten Einschränkungen an Volkshochschulen und dem Umgang damit veröffentlichte Matthias Rohs (2020) von der TU Kaiserslautern. Für diese Studie wurden im Mai und Juni 2020 in Zusammenarbeit mit dem rheinland-pfälzischen Landesverband der Volkshochschulen 10 Volkshochschulen in Rheinland-Pfalz mittels leitfadengestützter Telefoninterviews befragt (vgl. ebd., S. 5 f.). Die Ergebnisse der Studie zeigen, dass der pandemiebedingte Einbruch der Präsenzveranstaltungen „existenzielle Ängste“ (ebd., S. 29) bei den Volkshochschulen verursacht hat. Die Reaktionen der Volkshochschulen auf die Pandemie im zeitlichen Verlauf lassen sich in vier Phasen der Auseinandersetzung gliedern: 1. Schock-Phase (März), 2. Orientierung und Stabilisierung (Ende März bis Anfang April), 3. Kurzfristige Digitalisierung (April bis Mai) und 4. Systematische Flexibilisierung (Mai bis August/September) (vgl. ebd., S. 7–12; vgl. Maier und Rohs 2020, S. 39). Als zentrale Reaktion auf die Einstellung der Präsenzveranstaltungen zeigt sich die Digitalisierung der Angebote, wobei die Ausgangsbedingungen in den Einrichtungen mitunter voneinander abweichen (vgl. auch Sgodda 2021). Von den Befragten werden Chancen wie Risiken der Digitalisierung, wie bspw. die Erschließung neuer (überregionaler) Zielgruppen und der drohende Verlust von Stammpublikum, identifiziert. Auch diese besitzen je nach einrichtungsspezifischen Gegebenheiten in den Volkshochschulen unterschiedlich hohe Relevanz (vgl. Rohs 2020, S. 12–21, S. 30). Mit Blick auf die Digitalisierung der Volkshochschulen mitsamt ihren Angeboten benennt diese Studie die Pandemie als „Katalysator vorhandener Veränderungsbestrebungen“ (ebd., S. 20), wobei sich nicht alles Gewünschte am Ende in der Praxis umsetzen lasse und es daher entsprechender Abwägungen „zwischen dem technisch möglichen und den Rahmenbedingungen, unter denen die Volkshochschulen agieren“ (ebd., S. 27) bedürfe.

Grotlüschen und Weis (2021) haben Ergebnisse eines dreimonatigen Transferprojektes „Best Practices für neue Geschäftsmodelle und Programmanpassungen der Erwachsenenbildung“ an der Universität Hamburg vorgelegt. Sie befassten sich mit Veränderungs- und Planungsprozessen in verschiedenen Weiterbildungseinrichtungen in der Pandemie. Das Forschungsdesign umfasste 13 Interviews mit Personen, die primär im Feld der beruflichen Weiterbildung tätig sind. Deutlich wird aus Sicht der Befragten, dass Krisen nicht „top down“, sondern im Austausch bewältigt werden, wobei „Versuchsballons“ und Fehlertoleranz eine große Rolle spielen. Agilität wird mehr Aufmerksamkeit in der Programmplanung geschenkt (vgl. ebd., S. 53 f.).

Den Auswirkungen der COVID-19-Pandemie und des *Social Distancing* im Bereich Alphabetisierung und Grundbildung widmen sich Koppel und Langer (2020) von der PH Weingarten in vier leitfadengestützten Interviews. Die Gruppe der Befragten setzte sich zusammen aus Leitungspersonal von Alphabetisierungskursanbietern sowie Personen, „die in die Erstellung von grundbildungsbezogenen Konzeptionen an Bildungsinstitutionen involviert sind“ (ebd., S. 32). Die Interviews beschreiben Herausforderungen in der Praxis auf drei Ebenen. Auf konzeptioneller Ebene wird berichtet, dass ein „konzeptionell fundierter Einsatz digitaler Medien“ (ebd., S. 33) in der Praxis eine nachrangige Rolle einnimmt. Insbesondere der fehlende soziale Aspekt wird für Lernenden und Lehrenden als herausfordernd beschrieben, sodass häufig individuelle bilaterale Lösungswege in den Fokus rücken und vermehrt private Kommunikationswege wie Messenger-Dienste zum Einsatz kommen (vgl. ebd., S. 33 f.). Auf personeller Ebene stellen sich fehlender Zugang zu digitalen Medien sowie fehlende medienbezogene Kompetenzen der Lernenden als die zentralen Herausforderungen heraus. Sowohl bei Lernenden als auch Lehrenden wird von heterogenen Einstellungen gegenüber und Erfahrungen mit Online-Medien berichtet (vgl. ebd., S. 33). Auf institutioneller Ebene wird von einem Spannungsfeld zwischen dem erkannten Potenzial digitaler Medien und den mangelnden Möglichkeiten, diese im Kursalltag zu etablieren, berichtet. Die Ergebnisse zeigen, dass den Herausforderungen nur bedingt begegnet werden konnte (vgl. ebd., S. 34 f.).

#### Qualitative Studien in der Entstehung

Es ist anzunehmen, dass gegenwärtig weitere, in der Durchführung befindliche qualitative Forschungsprojekte Ergebnisse erbringen werden. Ein Beispiel ist das im April 2021 gestartete, 18-monatige Forschungsprojekt „Between educating and teaching the adult population. Andragogical perspectives on the Corona Pandemic“ unter der Leitung von Burkhard Schäffer, Denise Klinge und Arnd M. Nohl. Das Projekt wird von der Initiative „Corona Crisis and Beyond – Perspectives for Science, Scholarship and Society“ der VolkswagenStiftung gefördert (vgl. VolkswagenStiftung 2020). Es nimmt u. a. mittels narrativer Interviews das informelle Lernen der Bevölkerung via Medien und Politik in den Blick. Ein zweites Beispiel ist das Anfang 2021 gestartete und für zwei Jahre angesetzte Forschungsprojekt „Transformationsprozesse von öffentlichen Einrichtungen der Erwachsenen‑/Weiterbildung – Auswirkungen der Covid-19 Pandemie“ unter der Leitung von Maria Kondratjuk an der Technische Universität Dresden. Es folgt einem triangulativen Forschungsdesign aus problemzentrierten Interviews, Gruppendiskussionen, Dokumenten- sowie Institutionenanalysen.

## Erhebungen von Verbänden

Auch seitens der Weiterbildungsverbände wurden im ersten Jahr der COVID-19-Pandemie Erhebungen durchgeführt. Die Verbandsumfrage des Wuppertaler Kreis e. V. – Bundesverband betriebliche Weiterbildung wird jährlich durchgeführt. Im Jahr 2020 fokussiert sie die Situation der Weiterbildungsdienstleister in der COVID-19-Pandemie und zeigt, dass alle der aktuell 46 Mitgliedsunternehmen unmittelbar von der Krise betroffen sind. Knapp die Hälfte der Mitgliedsunternehmen erwartet Gesamtumsatzrückgänge von mehr als 20 % (vgl. Wuppertaler Kreis e. V. 2020, S. 2–4). Keine Veränderungen erwarten lediglich 8 %. Umsatzanstiege werden hingegen von allen Befragten ausgeschlossen. Eine vollständige Kompensation der Rückgänge durch digitale Angebote wird nicht erwartet. Neben den Umsatzrückgängen werden, bedingt durch die Maßnahmen zum Infektionsschutz, Entwicklungskosten für digitale Angebote, etc., deutliche Kostensteigerungen erwartet (vgl. ebd., S. 12 f.). Um Arbeitsplätze zu bewahren, hatten zum Zeitpunkt der Befragung bereits 51 % der Mitgliedsunternehmen Kurzarbeit angeordnet und z. T. andere Hilfs- und Unterstützungsprogramme genutzt (vgl. ebd., S. 7 f.). Laut der Verbandsumfrage führen die Auswirkungen der Pandemie zu einem Absinken des Geschäftslage-Indikators Weiterbildung von 118 auf 86 Punkte. Der Wert, der die wirtschaftliche Stimmung der Weiterbildungsbranche beschreibt, sinkt erstmals seit der Finanzkrise 2009 unter die 100 Punkte-Marke (vgl. ebd., S. 10). Weitere Schwerpunkte der Verbandsumfrage widmen sich den „Trends in der Weiterbildung“ (ebd., S. 15) sowie den „Veränderungen im Bereich der nach SGB geförderten Maßnahmen“ (ebd., S. 27).

Einen regionalen Blick auf Weiterbildungsanbieter nimmt die im April 2020 durchgeführte Blitzumfrage von Weiterbildung Hessen e. V. ein. An der Umfrage beteiligten sich 119 von über 300 Mitgliedsunternehmen. Im Fokus stehen hierbei die „Auswirkungen der COVID-19-Pandemie auf die hessischen Weiterbildungseinrichtungen“ (Weiterbildung Hessen e. V. 2020, S. 1). Die Ergebnisse der Blitzumfrage zeigen, dass sich die Pandemie negativ auf die finanzielle Situation der hessischen Weiterbildungseinrichtungen auswirkt. So berichten 89 % von Umsatzeinbußen und jede achte Einrichtung berichtet von einer drohenden Insolvenz. Eine zumindest anteilige Kompensation der Umsatzeinbußen durch Nachholeffekte erwarten lediglich 17 % der befragten Einrichtungen (vgl. ebd., S. 2 f.). Mit Blick auf die Beschäftigten der hessischen Weiterbildung wirkt sich die Pandemie zum Erhebungszeitpunkt in jeder zweiten Einrichtung vor allem auf die Honorarkräfte negativ in Form von Kündigungen bzw. ausbleibenden neuen Aufträgen aus. Pandemiebedingte Entlassungen Festangestellter kamen selten (3 %) vor. Diese Arbeitsplätze sollen vielmehr mit Hilfe staatlicher Unterstützungsleistungen (Kurzarbeitergeld) gesichert werden (vgl. ebd., S. 4). Zentrale Anpassungsstrategien der Einrichtungen an die neuen Gegebenheiten sind der verstärkte E‑Learning-Einsatz (62 %), der Ausbau der digitalen Infrastruktur (60 %) sowie ein steigender Social-Media-Einsatz bei der Kundenansprache (40 %). Problematisch ist, dass jede dritte Einrichtung mit einer unzureichenden Breitbandversorgung zu kämpfen hat (vgl. ebd., S. 6 f.). Als längerfristige Auswirkungen der COVID-19-Pandemie auf die Bildungslandschaft werden von den hessischen Weiterbildungseinrichtungen diese Punkte genannt: wachsende Anzahl an Insolvenzen, steigende Relevanz digitaler Angebote als Ergänzung zum Präsenzunterricht, verstärkter Einsatz von Social Media bei der Kundenansprache (vgl. ebd., S. 7).

## Analyse und Desiderate

Diese überblicksartige Darstellung zeigt, dass in der nationalen Weiterbildungsforschung bereits einige Studien zur COVID-19-Pandemie und deren Relevanz für die Weiterbildung vorliegen und weitere im Entstehen sind. Daneben gibt es noch zahlreiche weitere Publikationen und Dokumente aus der Wissenschaft, Praxis und Politik, die für die Erwachsenen- und Weiterbildung eine hohe Relevanz besitzen, da sie bspw. Befunde mit Lobbyarbeit verbinden oder maßgebliche Bestimmungen für die Erwachsenen- und Weiterbildung in der Pandemie festlegen. Ein Beispiel hierfür ist die Stellungnahme der Sektion Erwachsenenbildung der Deutschen Gesellschaft für Erziehungswissenschaft (DGfE) vom Juli 2020, die die Bedeutung der Weiterbildung in Krisenzeiten hervorhebt (DGfE 2020). Da der Fokus dieses Artikels auf dem wissenschaftlichen Forschungsstand liegt, wird von einer weitergehenden Aufzählung dieser Dokumente allerdings abgesehen.

Mit Blick auf die Analysefokusse der präsentierten nationalen Studien (s. auch Tab. 1) fällt auf, dass die Pandemie und ihre Auswirkungen vor allem mit Blick auf Institutionen und ihr Personal untersucht werden. Sowohl die quantitativen als auch die qualitativen Studien widmen sich im Kern den Auswirkungen der Pandemie auf entweder einzelne spezifische Anbieter von Weiterbildung wie Volkshochschulen (Rohs 2020), Betriebe (Flake et al. 2020; Bellmann et al. 2020), E‑Learning-Anbieter (Schmid und Goertz 2020), Anbieter von Alphabetisierungskursen (Koppel und Langer 2020) oder Weiterbildungsanbieter im Allgemeinen (Christ et al. 2021; Grotlüschen und Weis 2021). Auch die Verbände widmen sich in ihren Erhebungen den Auswirkungen der Pandemie auf Weiterbildungsanbieter in Form ihrer Verbandsmitglieder (Wuppertaler Kreis e. V. 2020; Weiterbildung Hessen e. V. 2020). Einen eher allgemeinen Blick auf die Effekte der Covid-19-Pandemie auf die Erwachsenenbildung liefert die international angelegte Delphi-Studie von Käpplinger und Lichte (2020). Der Faktor Personal bzw. die Effekte der Krise auf die bei den Anbietern beschäftigten Personen werden bspw. bei der Blitzbefragung des privaten mmb Instituts und der wbmonitor-Umfrage 2020 erhoben oder in den Interviews der Delphi-Studie thematisiert, wenngleich diese Thematik in den genannten Studien keinen eigenständigen Forschungsfokus darstellt. Mit Blick auf die fünf Forschungsfelder des Forschungsmemorandums für die Erwachsenen- und Weiterbildung (Arnold et al. 2000) lassen sich die veröffentlichten Studien überwiegend den Forschungsfeldern „Institutionalisierung“, „Professionelles Handeln“ sowie „System und Politik“ zuordnen, wobei, wie dargestellt, insbesondere die „organisatorische und institutionelle Wirklichkeit der Weiterbildung“ (ebd., S. 19) in den Fokus rückt. Im Forschungsfeld „Institutionalisierung“ stehen das „Betriebsgeschehen“ sowie „die Institutionalisierung lebenslangen Lernens und seiner unterschiedlichen Organisationsformen“ (ebd.) im Vordergrund. Das Forschungsfeld „Professionelles Handeln“ widmet sich der „Untersuchung von Berufstätigkeiten und Arbeitsanforderungen der Erwachsenenbildung und deren Veränderung“ (ebd., S. 15). Das Forschungsfeld „System und Politik“ fokussiert die strukturelle Ermöglichung von sowie strukturelle Fragen der Erwachsenen- und Weiterbildung (vgl. ebd., S. 24). Die weiteren Forschungsfelder des Forschungsmemorandums „Lernen Erwachsener“ sowie „Wissensstrukturen und Kompetenzbedarfe“ sind dagegen unterrepräsentiert. Dies bedeutet allerdings nicht, dass lediglich in diesen beiden Forschungsfeldern der Bedarf nach einer tiefergehenden und differenzierteren Beforschung der Auswirkungen der COVID-19-Pandemie zu konstatieren ist. Anhand der vorliegenden Recherche ergeben sich Forschungslücken in den nachfolgend aufgeführten sieben Punkten.

- *Weiterbildung in der Krise*
  Die Analysen zeigen einhellig, dass sich die Erwachsenen- und Weiterbildung 2020/2021 in einer Krise befand bzw. weiterhin befindet. Weiterbildungsaktivitäten sind deutlich zurückgegangen, wozu die Lockdowns und Hygienemaßnahmen wesentlich beigetragen haben. Allerdings wird auch mehrfach betont, dass Weiterbildungssegmente durchaus unterschiedlich stark betroffen sind. Hier erscheinen weitere Detailanalysen notwendig, um etwa genauer bestimmen zu können, wie sich das je nach Weiterbildungssegment oder Trägertyp, Beschäftigten- sowie Teilnehmendengruppe darstellt. In einigen Beiträgen finden sich Versuche, den Krisenverlauf in Phasen zu unterscheiden. Dies erscheint sinnvoll, da zum Beispiel zwischen dem ersten und zweiten Lockdown im Jahr 2020 mannigfache Unterschiede bestanden, wie in mehreren Studien erwähnt wird. Gleichzeitig steht es noch aus, eine genaue Phaseneinteilung vorzunehmen, was am besten mit zeitlichem Abstand retrospektiv erfolgen sollte. Was bislang ganz zu fehlen scheint, ist eine regional vergleichende Darstellung. Allein innerhalb Deutschlands stellten sich das Infektionsgeschehen und die Gegenmaßnahmen heterogen dar. Es konnten bspw. keine Studien gefunden werden, die regionale Vergleiche darüber hergestellt haben, wie das Weiterbildungsgeschehen im Detail mit Infektionsgeschehen und regionalen Gegenmaßnahmen interagiert.
- *Digitalisierungsschub*
  Die Studien kommen des Weiteren fast unisono zu dem Ergebnis, dass Corona zu einem massiven Digitalisierungsschub geführt hat, wenngleich auch Digitalisierungsprobleme sichtbar geworden sind (vgl. Schmidt-Hertha 2021). Dies gilt für alle Segmente der Weiterbildung, d. h. sowohl die betriebliche, berufliche als auch nicht-berufliche Weiterbildung. Kursleitungen wurden fortgebildet, um digital zu lehren. Weiterbildungseinrichtungen haben ihre digitale Infrastruktur optimiert. Planungsprozesse wurden verändert bzw. flexibel auf die Pandemielage angepasst. Es fällt auf, dass keine Studien gefunden werden konnten, in welchen evaluiert oder analysiert wurde, ob die neuen digitalen Lehr‑/Lernräume qualitativ erfolgreich waren. Sind die Lernenden und Stakeholder subjektiv mit den digitalen Lehr‑/Lernräumen zufrieden und wie stellen sich Lernerfolge dar? Hier kann eine erhebliche Notwendigkeit erkannt werden, den „Digital Boom“ mit Evaluations- und Wirkungsstudien zu begleiten und Beiträge zur Weiterentwicklung zu leisten.
- *Finanzierungsprobleme *
  Problematisch scheint, dass die neuen digitalen Angebote, oder auch weitere Versuche, auf die Krisenlage zu reagieren, nicht verhindern konnten, dass es zu massiven Einnahmeeinbrüchen in Weiterbildungseinrichtungen gekommen ist. In Berichten schildern Einrichtungsleitungen, dass finanzielle Rücklagen in der Krise aufgebraucht wurden oder sie ihre Zukunft problematisch einstufen. Bislang finden sich in den gesichteten Studien keine Hinweise zu Insolvenzen oder Geschäftsaufgaben in größerem Umfang. Ob dies so in den nächsten Monaten bleiben wird, muss untersucht werden. Interessant erscheinen Hinweise (vgl. Gnahs 2021), wie die Weiterbildung insgesamt „pandemiefest“ gemacht werden könne und wie strukturelle Probleme der Weiterbildung, wie die Unterfinanzierung, angegangen werden könnten.
- *Organisationale und programmplanerische Veränderungen*
  Ähnliches gilt für neue, veränderte Planungs- und Organisationsprozesse, die mehr Agilität und Telearbeit miteinbeziehen. Die genauen Auswirkungen auf die Organisationsentwicklung und Programmplanung können bislang nur erahnt werden. Einige Einrichtungen haben (oft aus Kostengründen) gedruckte Programme eingestellt und auf rein digitale Programme umgestellt (Rohs 2020, S. 4). Es ist nicht absehbar, ob das eine akute Krisenmaßnahme ist oder von Dauer sein wird. Interessant sind Entwicklungen von neuen Kursbuchungsplattformen u. a. auf Verbandsebene (Klemm und Repka 2021). Generell besteht eine Notwendigkeit, diese Entwicklungen mit kritischer Distanz zu analysieren. In der Organisationsberatung kann Agilität leicht normativ und Slogan-artig überhöht werden.
- *Informelles Lernen*
  Bisher wenig untersucht erscheint die Rolle des informellen Lernens in der Pandemie, wenngleich Expertinnen und Experten auf dessen Bedeutung hinweisen (Käpplinger und Lichte 2020, S. 784). Kaum jemand dürfte in der Pandemie einen Kurs in der organisierten Weiterbildung zu den AHA-Regeln oder zur Interpretation von Infektionszahlen besucht haben. Trotzdem haben die Menschen in den Jahren 2020 und 2021 viel über Corona gelernt – und dies eher en passant aus staatlichen Verordnungen und aus ihrem privaten oder beruflichen Umfeld und nicht zuletzt durch öffentlich-rechtliche sowie soziale Medien. Es sei nur exemplarisch an den NDR-Podcast des Virologen Christian Drosten erinnert, der Kultstatus erreichte. Dieser Podcast kann als eine Art Erwachsenenbildung begriffen werden. Es könnte ein Ergebnis sein, dass die Weiterbildungsberichterstattung im AES 2020 von einem Einbruch der kursförmigen Teilnahme berichten, während die Weiterbildungsteilnahme im Jahr 2020 insgesamt durch digitale Schulungs- und Informationsformate angestiegen sein wird.
- *Medienbildung und Politische Bildung*
  Allerdings zeigt das informelle Lernen in der Pandemie die Problematik informellen Lernens auf. Im Zeitraum 2020/2021 gab es auch eine Pandemie der Fake News. Gerade in den sozialen Netzwerken können Dinge gelernt werden, die nicht der Wahrheit entsprechen. Die öffentlich-rechtlichen Medien müssen sich gleichzeitig mit Medienanalysen reflexiv befassen, die kritisch eine „Verengung der Welt“ (Gräf und Hennig 2020) feststellen, wo Corona angesichts anderer Probleme in der Welt zu viel Aufmerksamkeit geschenkt wurde. Zeithistoriker stellen nüchtern die Prognose, dass noch geklärt werden müsse, ob es sich bei COVID-19 um eine „skandalisierte Krankheit“ (Fangerau und Labisch 2020) handele, da die Krankheit zweifellos schlimm sei, aber die Art und Weise der Dramatisierung nicht mehr verhältnismäßig sein könne. Barz (2021) thematisiert die Frage der Risikokompetenz am Beispiel von Corona. Letztlich scheinen bei diesen Themen zwischen Fake und Fakten kaum differenzierte Diskussionen möglich zu sein. Gleichwohl gehört es zu den Aufgaben der politischen Bildung, in erhitzten Diskursen zur Versachlichung und zu einer Debatte über Meinungsunterschiede hinweg beizutragen. Dies wird in post-pandemischen Zeiten wahrscheinlich eine bedeutsame Aufgabe sein.
- *Perspektive der Lernenden*
  Was Corona und die Gegenmaßnahmen mit den Lernenden und Zielgruppen „gemacht“ hat, kann als Forschungslücke benannt werden. Welche Weiterbildungsbedürfnisse bestehen in der Pandemie? Wie wurde mit digitalem Lernen (vgl. Dinkelaker 2021) umgegangen? Erhebungen zur sogenannten „Zoom-Fatigue“ liegen vor (z. B. Rump und Brandt 2021), sind aber nicht erwachsenenpädagogisch rückgebunden worden. Welche Lerngruppen hatten in der Pandemie mit Benachteiligungen zu tun? Kam es zu einer Re-Traditionalisierung der Geschlechterrollen (Kohlrausch und Zucco 2020; Krohn 2020) im Kontext der Weiterbildung? Welche neuen Formen von Lernen und Lernzeitarrangements haben sich entwickelt, die sogar ein Mehr an Inklusion ermöglichen? Inwiefern waren Arbeiten und Lernen von Zuhause aus während der Lockdowns möglich? Sind neue Lernbedarfe an der Grenze zur Therapie entstanden?

## Fazit

Die in diesem Artikel vorgestellten Studien bieten einen Überblick über die bestehenden sowie zu erwartenden Effekte der COVID-19-Pandemie auf die Erwachsenen- und Weiterbildung. Dies schafft eine erste Grundlage, um sich in weiteren Forschungen tiefergehender, differenzierter und auch langfristiger mit COVID-19 und den daraus erwachsenen Veränderungen auseinanderzusetzen. Die herausgearbeiteten Desiderate verdeutlichen, dass ein erheblicher Forschungsbedarf hinsichtlich der Auswirkungen der COVID-19-Pandemie auf die Erwachsenen- und Weiterbildung besteht.

Es gibt Ansätze in der gesichteten Literatur, diese Pandemie in Phasen zu unterteilen. Dies erscheint sinnvoll, wenngleich eine tragfähige Einteilung wohl erst nach der Pandemie vorgenommen werden kann. Insgesamt zeigt sich die COVID-19-Pandemie als Disruptor für die Erwachsenen- und Weiterbildung, durch den viele bisherige Aktivitäten unter- bzw. abgebrochen wurden. Gleichzeitig war die Krise ein Katalysator für Neu- und Weiterentwicklungen. Dies gilt insbesondere für den Bereich des digital gestützten Lehrens und Lernens. Digitalität ist nicht nur eine technische Frage, sondern eng mit weiteren Fragen rund um Organisationsentwicklung, Programmplanung und Personalentwicklung sowie Gerechtigkeitsfragen (Sturm 2021) verwoben. Es wäre eine irrige Annahme zu behaupten, dass Lehr‑/Lernkulturen aus der physischen Präsenz vor Ort in die Digitalität übertragen werden können.

Aber nicht alles ist neu seit bzw. durch Corona. Gerade die zu beobachtenden Probleme – wie die Prekarität bestimmter Beschäftigungssegmente (Probst 2020) oder die chronische Unterfinanzierung in der Weiterbildung – sind bekannte Phänomene, die nun wie unter einem Brennglas umso schärfer zu Tage treten und ins Bewusstsein drängen. Wenn man die COVID-19-Krise als Anlass dafür nähme, diese Probleme strukturell anzugehen, könnte in der Krise eine Chance liegen (vgl. Luibl 2020).

Abschließend soll an dieser Stelle auf die Limitationen des hier vorgestellten Forschungsüberblicks hingewiesen werden. Diese Zusammenschau stellt eine Momentaufnahme dar. Es kann selbst in diesem Moment, in dem wir das Manuskript in den Verlag verabschieden, nicht gesichert behauptet werden, dass alles recherchiert und aufgenommen werden konnte, was derzeit im Entstehen ist. Jede Analyse braucht zeitlichen Abstand. Nichtsdestoweniger hoffen wir, für weitere Forschungen eine Basis zur Verfügung zu stellen, indem wir auf Desiderate hingewiesen und Impulse für neue Forschungsfragen gegeben haben.

## Footnotes

1 Zeitschrift für Pädagogik, Zeitschrift für Erziehungswissenschaft, Zeitschrift für Weiterbildungsforschung, Hessische Blätter für Volksbildung, Weiterbildung (ehemals GdWZ), weiter bilden, Der Pädagogische Blick, Forum Erwachsenenbildung, Volkshochschulen in Berlin, RELA und International Review of Education.

2 Sektion Erwachsenenbildung der DGfE, ESREA – European Society for Research on the Education of Adults, CASAE – Canadian Association for Adult Education, CPAE – Commission of Professors of Adult Education, RWL – Research Conference on Work and Learning.

3 Wir legen hier ein eher weites Verständnis an, sodass u. a. auch nicht-universitäre Forschung erfasst wird. Es wurden dabei auch Studien von Instituten aufgenommen, die Interessenorganisationen (Arbeitgeber, Praxisverbände, etc.) nahestehen oder von diesen finanziert werden.

4 International befasst sich bspw. der Schweizerische Verband für Weiterbildung in seinem Branchenmonitor mit pandemiebedingten Entwicklungen auf dem Schweizer Weiterbildungsmarkt (vgl. Poopalapillai et al. 2021). Weitere internationale Beiträge finden sich u. a. in zwei Spezialausgaben des International Review of Education mit den Titeln „Education in the age of COVID-19: Implications for the future (2021)“ und „Education in the age of COVID-19: Understanding the consequences (2020)“ vielfältige pandemiebezogene Beiträge (Béché 2020; Bjursell 2020; Eschenbacher und Fleming 2020; Galloway et al. 2020; Green et al. 2020; Landa et al. 2021; Lyu et al. 2020; Seetal et al. 2021; Smythe et al. 2021; Stack 2021; Stanistreet et al. 2020, 2021; Zaalouk et al. 2021).
